# Forming quasicrystals by monodisperse soft core particles

**DOI:** 10.1038/s41467-017-02316-3

**Published:** 2017-12-12

**Authors:** Mengjie Zu, Peng Tan, Ning Xu

**Affiliations:** 1 CAS Key Laboratory of Soft Matter Chemistry, Hefei National Laboratory for Physical Sciences at the Microscale, and Department of Physics, University of Science and Technology of China, Hefei, 230026 People’s Republic of China; 2 State Key Laboratory of Surface Physics and Department of Physics, Fudan University, Shanghai, 200433 People’s Republic of China

## Abstract

In traditional approaches to form quasicrystals, multiple competing length scales involved in particle size, shape, or interaction potential are believed to be necessary. It is unexpected that quasicrystals can be formed by monodisperse, isotropic particles interacting via a simple potential that does not contain explicit multiple length scales to stabilize quasicrystals. Here, we report the surprising finding of the formation of such quasicrystals in high-density systems of soft-core particles. Although there are length scales naturally introduced in our model systems, they do not establish the quasicrystalline order. In two dimensions, we find not only dodecagonal but also octagonal quasicrystals, which have not been found yet in soft quasicrystals. In such unexpected quasicrystals, particles tend to form pentagons, which are essential elements to develop the quasicrystalline order. Our findings thus pave an unexpected and simple way to form quasicrystals and pose a challenge for theoretical understanding of quasicrystals.

## Abstract (web summary)

Quasicrystals with structural symmetries forbidden in crystals have been found in alloys or mono-component systems composed of anisotropic units. Zu et al. show a formation of two-dimensional quasicrystals in an isotropic soft solid with a spring-like potential, which challenges the existing theory.

## Introduction

Quasicrystal (QC) is a fantastic discovery in materials science and condensed matter physics^1,2^, which exhibits a rotational symmetry forbidden in periodic crystals. Since the first observation of a decagonal QC in Al–Mn alloys^1^, thousands of metallic QCs have been obtained^3^. These QCs intrinsically involve multiple length scales arising from multi-type atoms.

Soft or mesoscopic (non-metallic) QCs have brought great attention to the community of QCs recently^4–9^, since the first finding of a 12-fold QC in supramolecular dendrimers^10^. Compared with metallic QCs, soft materials have displayed advantages in forming stable mono-component QCs. However, to purposely introduce multiple QC-favored length scales still seems to be inevitable to form soft QCs^11^. Until now, soft QCs were obtained by either introducing multiple competing length scales in the inter-particle potential chosen in specific ratios to favor QC formation^12–17^ or using anisotropic particles naturally possessing multiple length scales, such as tetrahedral and patchy particles^18,19^. As far as we know, there has been no report yet about QCs formed by mono-component, isotropic particles interacting via a smooth potential that does not explicitly involve characteristic length scales to stabilize the QCs.

Here, we show that such unexpected formation of soft QCs does exist in high-density systems consisting of monodisperse, soft-core particles interacting via a simple spring-like pairwise repulsion. We observe both octagonal and dodecagonal QCs (OQCs and DDQCs) and find that the particles spontaneously form pentagons, which are essential elements in the formation of our QCs.

## Results

### Phase diagram of solid states

The inter-particle potential is $U(r) = \frac{\epsilon }{\alpha }\left( {1 - r{\mathrm{/}}\sigma } \right)^\alpha {{\Theta }}\left( {1 - r{\mathrm{/}}\sigma } \right)$, where *r* is the separation between two particles, *σ* is the particle diameter or range of interaction, $\epsilon$ is the characteristic energy scale, *α* determines the softness of the potential, and Θ(*x*) is the Heaviside step function. To avoid the clustering of particles^20,21^, we vary *α* from 2.0 to 3.0. In the main text here, we focus on two-dimensional systems. In Supplementary Fig. 1 and Supplementary Note 1, we also show and discuss some preliminary results of three-dimensional systems.

**Fig. 1:**
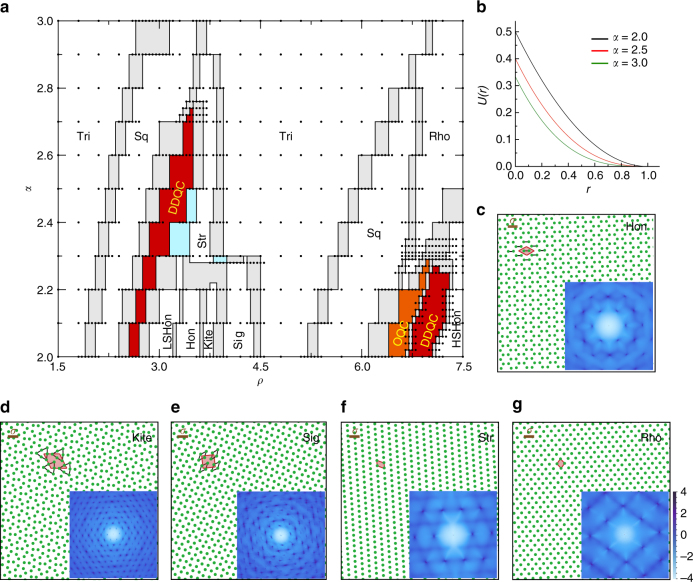
Multiple solid phases formed by soft-core particles at high densities. **a** Phase diagram of solid states in terms of number density *ρ* and potential exponent *α* at a fixed temperature *T* = 10^−4^. The black dots label the (*ρ*, *α*) pairs where we run simulations to identify states. The orange and red areas are the territories of OQCs and DDQCs, respectively. The gray and light-blue areas are phase coexistence regimes of two and three types of solids, respectively. In addition to the abbreviations defined in the text, LSHon and HSHon denote lower- and higher-density stretched honeycomb, respectively. **b** Examples of particle interaction potentials with different *α*. **c**–**g** A part of static configurations of five distinct crystalline solids with the scale bar indicating the actual size of particle diameter *σ*. The (*ρ*, *α*) values of the five states are (3.35, 2.0), (3.70, 2.0), (3.95, 2.0), (3.60, 2.3), and (6.90, 2.4), respectively. The red-shaded polygons and black lines outline the unit cell and basis, respectively. The insets are diffraction patterns with the values of the scale bar being in the logarithmic scale

With increasing number density *ρ* at fixed temperature *T*, solid phases with different structures emerge in sequence, as shown in Fig. 1a. Figure 1b shows that the inter-particle potential does not exhibit multiple length scales. Surprisingly, in certain (*ρ*, *α*) parameter regimes, both OQCs and DDQCs appear. To our knowledge, OQCs have not yet been convincingly observed in soft QCs.

In Fig. 1c–g, we show the static configuration and diffraction pattern (characterized by the static structure factor as defined in the Methods section) of five special crystals other than the ordinary triangular (Tri) and square (Sq) solids, including honeycomb (Hon), kite (Kite), sigma-phase (Sig), stripe (Str), and rhombus (Rho) solid. Each solid has a definite unit cell and basis as outlined in the configuration. Although some unit cells are complicated, they repeat periodically in space, leading to a periodic diffraction pattern.

### Structure and dynamics of QCs

In the (*ρ*, *α*) parameter space studied here, QCs exist in three isolated regimes. OQCs occupy a regime with small *α* and high *ρ*. DDQCs emerge in two regimes: one adjacent to OQCs (higher-density DDQC (HDDQC)) and the other at relatively low *ρ* (lower-density DDQC (LDDQC)), covering a wider range of *α*.

**Fig. 2:**
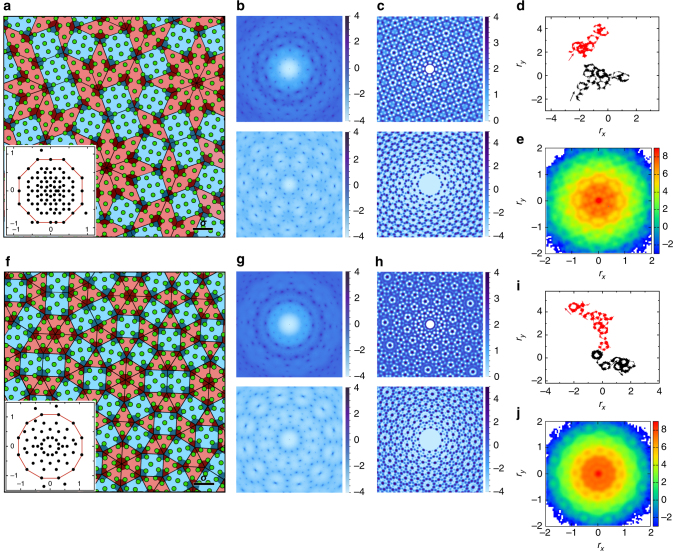
Characterization of the structure and dynamics of two types of quasicrystals. **a**–**e** and **f**–**j** are for an OQC and a DDQC at (*ρ*, *α*, *T*) = (6.6, 2.0, 1.36 × 10^−3^) and (7.0, 2.0, 1.72 × 10^−3^), respectively. **a**, **f** A part of static configuration with the square–rhombus (square–triangle) tiling. The black scale bar indicates the actual size of particle diameter *σ*. Note that the shaded pentagons prevail, whose centers are connected to construct the tessellation. The inset shows the projection of the QC in the perpendicular space with the red polygon being the atomic surface. **b**, **c**, **g**, **h** Diffraction patterns and density profiles calculated from single particles (top panel) and from pentagons (bottom panel). **d**, **i** Particle trajectories during a time interval of 10^5^ with the arrows pointing to the original direction of motion. **e**, **j** van Hove autocorrelation function *G*_a_(*r*, *t*) at *t* = 6000. The values of the scale bars are in the logarithmic scale

Figure 2a shows a part of static configuration of an OQC. By a cursory look, we can identify many octagons and pentagons. The diffraction pattern shown in the top panel of Fig. 2b contains discrete sharp Bragg peaks with an eight-fold symmetry, similar to that of the OQC of Cr–Ni–Si alloys^22^. The density profile (characterized by the inhomogeneous radial distribution function as defined in the Methods section) shown in the top panel of Fig. 2c further confirms the symmetry and the loss of density periodicity.

The rotational symmetry and quality of a QC can also be detected from the structure of particle (cluster center) projections in the perpendicular space orthogonal to the physical space where the QC resides^23,24^ (see details of the calculation in the Methods section). The perpendicular space shown in the inset of Fig. 2a exhibits the same symmetry as observed in the physical space. We find that about 99.8% of the particles are projected within an octagon named as the atomic surface^23^, indicating that the configuration shown in Fig. 2a is indeed an almost perfect OQC.

A closer look at Fig. 2a reveals that each pentagon is surrounded by eight particles, which form a nice octagon. This implies that pentagons may be important structural elements in forming OQCs in our systems. Here we employ a polygonal order parameter $\delta = {\mathrm{max}}\left\{ {\left| {e_i{\mathrm{/}}\bar e - 1} \right|} \right\}$ (*i *= 1, 2, ..., 5) to numerically identify pentagons, where *e*_*i*_ is the distance between the center of mass and vertex *i* of a 5-sided polygon, and $\bar e = \mathop {\sum}\nolimits_{i = 1}^5 e_i{\mathrm{/}}5$. Only 5-sided polygons with *δ* < 0.1 are identified as pentagons. By connecting the centers of non-edge-adjacent pentagons, Fig. 2a shows that the OQC can be tessellated by 45° rhombi and squares. The number ratio of squares to rhombi is approximately 0.701, close to $1:\sqrt 2$ for perfect OQCs^25^. As shown in the bottom panel of Fig. 2b, when plotting the diffraction pattern based on centers of pentagons, the Bragg peaks become much sharper than those for single particles. Therefore, better quasicrystalline order is achieved by pentagons.

In addition to structures, the quasicrystalline order and significance of pentagons can be further verified from dynamics. Figure 2d shows the trajectories of two randomly chosen particles in the OQC. The trajectories are composed of a chain of pentagon loops. A particle moves along the edges of a pentagon for a long time and suddenly escapes from the pentagon and forms a new pentagon with other particles, corresponding to a phason flip, whose presence causes liquid-like diffusion in QCs^26–28^ (see Supplementary Movies 1 and 2, Supplementary Note 2, and Supplementary Fig. 2 for more information about the dynamics). The pentagon loops are special for our QCs, which further emphasizes the importance of pentagons.

The rotational symmetry of the OQC can be seen as well from the van Hove autocorrelation function *G*_a_(**r**, *t*) (Fig. 2e; defined in the Methods section), which quantifies the probability distribution that a particle has been displaced by **r** at time *t*. In an intermediate time regime (*t* = 6000 here), particles exhibit clear heterogeneous displacements (see Supplementary Fig. 2 for the time evolution of *G*_a_(**r**, *t*)). There are particles vibrating around their equilibrium positions, forming the central peak at **r** = 0. Surrounding the central peak are satellite peaks with an eight-fold symmetry, consistent with the QC symmetry shown in the structure.

Figure 2f–j shows the same structural and dynamical information for a HDDQC (structural information of LDDQCs can be seen in Supplementary Note 3 and Supplementary Fig. 3). Interestingly, pentagons are still remarkable. As shown in Fig. 2f, each pentagon is surrounded by 12 particles sitting on the vertexes of a dodecagon. Again, by connecting the centers of non-edge-adjacent pentagons, the whole DDQC can be tiled by squares and triangles. The number ratio of triangles to squares is about 2.283, close to the ideal value of $4{\mathrm{/}}\sqrt 3$ for perfect DDQCs^29^.

Note that we can tessellate our OQCs by squares and rhombi on both the single-particle and pentagon levels. However, for DDQCs, we can only realize the square–triangle tiling based on pentagons. Therefore, pentagons are indispensable in characterizing our DDQCs. This significantly highlights the importance of pentagons in our QCs.

### Length scales

A limitation of our QCs is the lack of explicit characteristic length scales to establish the quasicrystalline order. There are length scales naturally introduced in our model systems, e.g., average nearest particle separation and potential cutoff. Note that these intrinsic length scales exist in most of the model systems, but only a small number of purposely designed systems can form QCs. In this section, we will discuss about the characteristic length scales in our QCs and the roles of intrinsic length scales in the formation of our QCs.

**Fig. 3:**
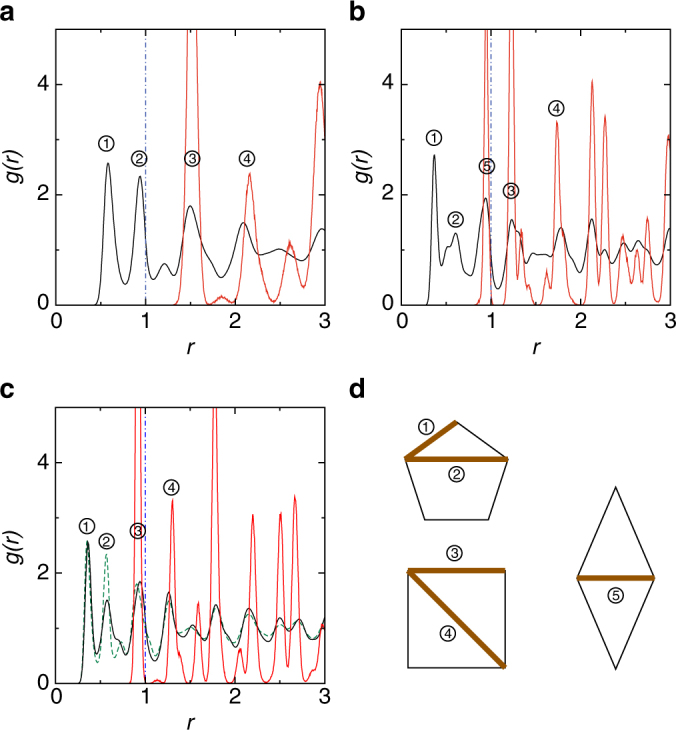
Comparison of radial distribution functions *g*(*r*) of QCs for *α* = 2.0. **a**–**c** are for LDDQC, OQC, and HDDQC at (*ρ*, *T*) = (2.56, 1.53 × 10^−3^), (6.60, 1.36 × 10^−3^), and (7.00, 1.72 × 10^−3^), respectively. The black and red solid curves are calculated based on particles and centers of pentagons, respectively. Note that the major peaks of the red curves match well with those of the black curves. The green dashed curve in **c** is *g*(*r*) of LDDQC after *r* is divided by (7.00/2.56)^1/2^ ≈ 1.6536, which shows identical peaks to *g*(*r*) of HDDQC. The vertical dot–dashed lines mark the location of the potential cutoff at *r* = 1. **d** Schematic plots of the characteristic length scales in QCs. The pentagon sketches the basic structural element formed by five particles. The square and rhombus sketch the polygons in the square–triangle and square–rhombus tilings by connecting centers of pentagons. The numbers 1–5 denote the side length of pentagons, diagonal length of pentagons, side length of squares, diagonal length of squares, and shorter diagonal length of rhombi, respectively. Note that in the tilings the equilateral triangles and rhombi have the same side length as the squares, so length no. 3 actually represents the side length of all polygons in the tilings. In **a**–**c**, we number the peaks corresponding to the length scales

In Fig. 3a–c, we show the radial distribution functions *g*(*r*) of LDDQC, OQC, and HDDQC. For each QC, we separately calculate *g*(*r*) for particles and for centers of pentagons. There are peaks in both sets of *g*(*r*), representing different length scales in the QC. We identify the typical length scales in our QCs, as sketched in Fig. 3d. These lengths are the shortest ones to form the basic structural element (pentagon) and establish the quasicrystalline order.

For all QCs, the first two peaks in *g*(*r*) calculated for particles correspond to the side and diagonal lengths of pentagons, which are also the two nearest particle separations. These two length scales are essential in the formation of pentagons, but they are not the lengths to establish the long-range quasicrystalline order. Their emergence cannot be straightforwardly predicted from our knowledge about normal uniform systems and thus remains elusive.

The basic length scales to establish the quasicrystalline order can be identified from the polygon tessellation of the QCs, e.g., Fig. 2a, f. For DDQCs, they are the side length of squares and equilateral triangles and the diagonal length of squares, which correspond to the first two peaks in *g*(*r*) calculated for centers of pentagons. These two peaks coincide with the third and forth peaks in *g*(*r*) calculated for particles, as shown in Fig. 3a, c. Therefore, from the perspective of particles, the third and fourth peaks in *g*(*r*) represent length scales to establish the quasicrystalline order. For OQC, the basic lengths to establish the quasicrystalline order are the shorter diagonal length of rhombi, side length of squares and rhombi, and diagonal length of squares. They correspond to the first three peaks in *g*(*r*) calculated for centers of pentagons and coincide with the third, fourth, and fifth peaks in *g*(*r*) calculated for particles, as shown in Fig. 3b.

Figure 3 also indicates that the potential cutoff should not act as a necessary length for our QCs. The potential cutoff represents rather different length scales for LDDQC and HDDQC. As can be seen from the configurations (Fig. 2f and Supplementary Fig. 3a), HDDQC and LDDQC have similar structures with the same square–triangle tessellation. When particle separation *r* is scaled by density, *g*(*r*) values of LDDQC and HDDQC exhibit identical peaks, as shown in Fig. 3c. Therefore, if the potential cutoff was an essential length scale, it would play the same role in both DDQCs. However, this is not the case. The ratio of the potential cutoff to the side length of pentagons [corresponding to the first peak of *g*(*r*) calculated for particles] is obviously quite different for the two DDQCs. Moreover, there are two peaks in *g*(*r*) calculated for particles before the potential cutoff for LDDQC, while there are three for HDDQC. Since the two DDQCs have the same set of length scales in *g*(*r*) but the relative locations of the potential cutoff are quite different, the potential cutoff should have nothing to do with our QCs, although it does exist as an intrinsic length scale in model systems.

### Signs of QC formation in liquids

All solid states shown in Fig. 1a are obtained by slowly quenching liquids below the melting temperature *T*_m_. It has been proposed that prior to freezing some local order may have already developed in liquids^30^. Since pentagons are essential in our QCs, one may wonder whether they can be tracked in liquids. Moreover, as discussed above, it remains mysterious how the characteristic length scales spontaneously emerge to stabilize the QC order. Searching for competing length scales in liquid states prior to the phase transition to QCs may provide us with some clues.

**Fig. 4:**
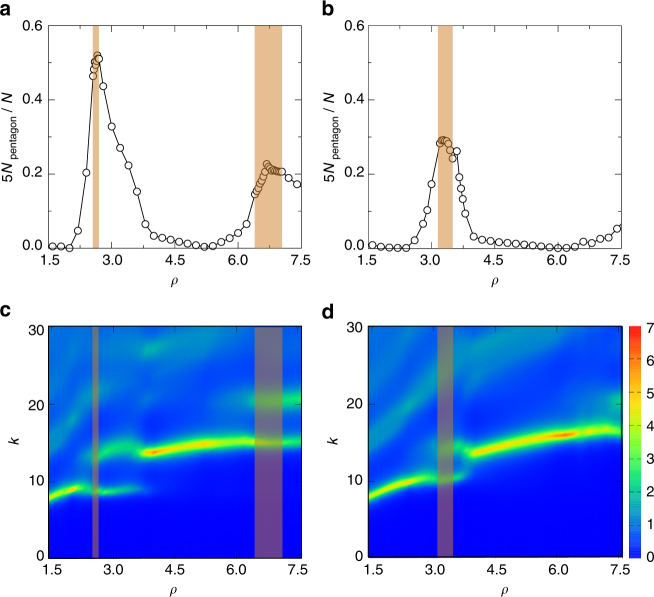
Structural information in liquids. The left and right columns are for *α* = 2.0 and 2.5, respectively. **a**, **b** Density dependence of the fraction of particles forming pentagons, 5*N*_pentagon_/*N*. **c**, **d** Contour plots of the static structure factors *S*(*k*) with the color bar quantifying the values of *S*(*k*). The liquids characterized here are slightly above the melting temperature at *T* = 1.1*T*_m_. The shadowed density regimes in brown are where QCs exist

We thus compare the structures of liquids at *T* = 1.1*T*_m_ over the whole range of densities of Fig. 1. The temperature envelop slightly above *T*_m_ chosen here assures that the liquids stay at approximately the same distance away from the establishment of (quasi)crystalline order. In Fig. 4, we show the density dependence of the fraction of particles forming pentagons, 5*N*_pentagon_/*N*, and static structure factor, *S*(*k*), for the liquids with harmonic (*α* = 2.0) and Hertzian (*α* = 2.5) repulsions, where *N*_pentagon_ and *N* denote the number of pentagons and total number of particles (see Supplementary Fig. 4 and Supplementary Note 4 for the evolution of 5*N*_pentagon_/*N* across the liquid–solid transition). Figure 4a, b indicates that pentagons have already accumulated in QC-forming liquids, leading to the maxima in 5*N*_pentagon_/*N*. The contour plots of *S*(*k*) in Fig. 4c, d demonstrate two pronounced low-*k* peaks in the density regimes where QCs reside.

Note that our systems are at high densities. With increasing density, each particle can interact with more and more neighbors, leading to repeated emergence of the same types of solids, e.g., triangular solid^31,32^. Referring to Fig. 1a, the two low-*k* peaks in *S*(*k*) are apparently associated with the first peak (representing the average nearest particle separation) of the liquids forming the two triangular solids on the lower- and higher-density sides of QCs. However, these two competing length scales are not unique for QC-forming liquids. They may provide a necessary (but not sufficient) condition for our QCs to occur.

Since pentagons already accumulate in liquids, the emergence of the two competing length scales indicates that two nearest particle separations characterizing pentagons are developed. The ratio of the two *k* values for the peaks in *S*(*k*) is indeed close to the ratio of the diagonal length to the side length of a pentagon. Therefore, these emerging length scales are important to construct the basic element of our QCs, but they do not establish the quasicrystalline order, as discussed above. More in-depth studies are required to find out the origin of these lengths and their roles in the spontaneous formation of the quasicrystalline order, while the key is to sort out the complicated interplay between high density and soft-core potentials.

### Stability of QCs

**Fig. 5:**
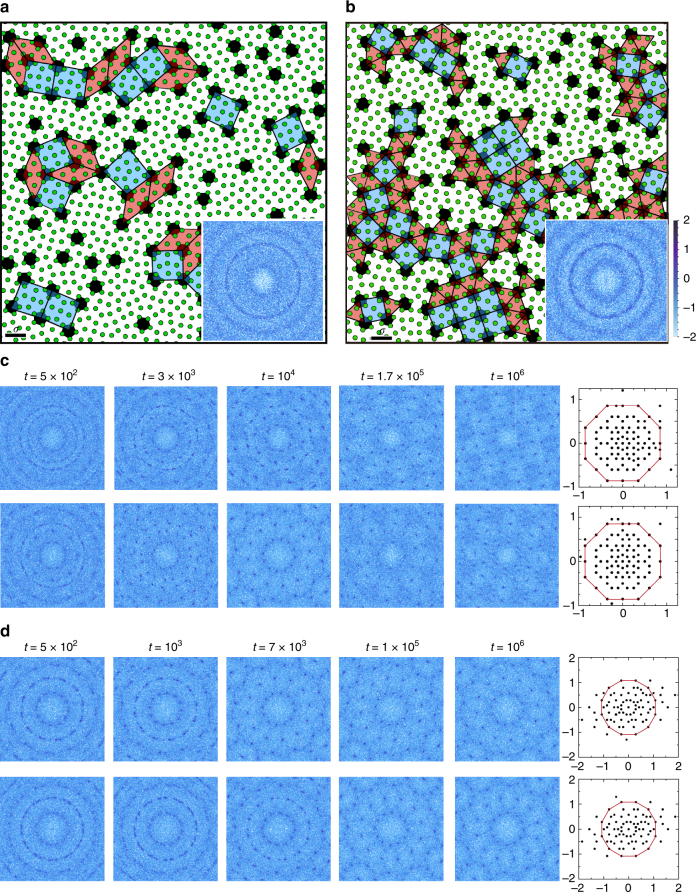
Verification of route independence of the QC formation for systems with *α* = 2.0. **a**, **b** *T* = 0 configurations obtained by quickly quenching ideal gas states to local potential energy minima at *ρ* = 6.50 and 6.80, respectively, where OQCs and DDQCs are supposed to exist. The black scale bars indicate the actual size of particle diameter *σ*. The squares, rhombi, and triangles are characteristic polygons to tessellate QCs, which are identified by connecting the centers of non-edge-adjacent pentagons. The insets are diffraction patterns of the *T* = 0 configurations with the scale bar showing values in the logarithmic scale. **c**, **d** Time evolution of the diffraction patterns and projection of the long-time relaxed state in the perpendicular space (the rightmost panel) at (*ρ*, *T*_f_) = (6.50, 1.27 × 10^−3^) (OQC) and (6.80, 1.49 × 10^−3^) (DDQC) respectively, along two different routes. The initial states of the top and bottom panels are *T* = 0 states shown in **a** and **b** and equilibrated states at *T* > *T*_m_, respectively. Both types of states are suddenly brought to *T* = *T*_f_ at *t* = 0. The red polygons in the perpendicular space are the atomic surface. All diffraction patterns are calculated based on centers of pentagons

Now there comes a question whether our QCs are stable. In order to verify the stability of our QCs, in addition to slow quenching, we examine the formation of QCs along another two different routes at a fixed density. In the first route, we quickly quench an ideal gas state to a local potential energy minimum to get the *T* = 0 state. Then we suddenly increase the temperature to *T*_f_ where QCs exist. In the second route, we equilibrate a liquid at *T* > *T*_m_ and then suddenly decrease the temperature to *T*_f_. For both cases, we observe the time evolution of the structures at fixed *ρ* and *T* = *T*_f_, as shown in Fig. 5.

Figure 5a, b shows the quickly quenched *T* = 0 configurations at *ρ* = 6.50 and 6.80 for harmonic repulsion, where OQCs and DDQCs should exist. We can still identify some pentagons. By connecting the centers of non-edge-adjacent pentagons, we can partially tessellate the configurations using the QC tessellation. Therefore, the *T* = 0 configurations are random tilings of QC polygons. The diffraction patterns based on centers of pentagons contain nearly isotropic rings and do not exhibit QC symmetries. In Fig. 5c, d, we compare the time evolution of the diffraction patterns along two routes at (*ρ*, *T*_f_) = (6.50, 1.27 × 10^−3^) and (6.80, 1.49 × 10^−3^). We can clearly see the formation of the same QC symmetry along both routes. To further illustrate the symmetry and quality of the QCs, we also show in Fig. 5c, d the projection of the long-time relaxed state in the perpendicular space. Most of the particles (cluster centers) are projected within the atomic surface, exhibiting a nice eight- or 12-fold rotational symmetry. The projections of the states along two different routes show similar patterns. Therefore, the QCs reported here are stable and independent of history. Probably because the systems have not been relaxed for enough long time, there are still a few spots outside the atomic surface, leading to an elongated distribution as shown in Fig. 5d. The patterns in the perpendicular space may evolve to that shown in the inset of Fig. 2f (obtained by slow quenching) if the systems are relaxed for sufficiently long time.

**Fig. 6:**
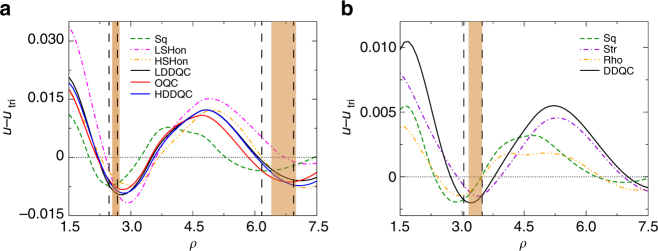
Comparison of the potential energies of the zero temperature QCs and neighboring solids. **a** and **b** are for *α* = 2.0 and *α* = 2.5, respectively. The potential energy per particle *u* is subtracted by that of a perfect triangular lattice, *u*_tri_. The horizontal dotted line marks *u* = *u*_tri_. The density regimes demarcated by the vertical dashed lines and brown bands are where QCs have the lowest potential energy and where QCs exist, respectively. The absence of perfect match between the two may be due to phase coexistence

Figure 6 provides another evidence of the QC stability. There we further compare the *T* = 0 potential energies of QCs with those of crystalline solids next to them for harmonic and Hertzian repulsions. For each type of the solids, we fix the locations of particles and calculate the potential energy (sum of particle interaction potential) as a function of density by varying the diameter of particles. In the density regimes where we find QCs, the corresponding QCs have the lowest potential energy. Because the structures of QCs are more random than those of crystals, it is plausible to assume that the entropy of thermal QCs is higher as well. Thus, at *T* > 0, QCs should have a lower free energy than other crystals and are stable enough to survive.

## Discussion

The most surprising aspect of this work is the finding of a new class of soft QCs with complex structural units in such simple systems without any explicit multiple characteristic length scales required to stabilize the QCs. Although there are intrinsic length scales in our systems, they do not stabilize our QCs. According to existing theories, QCs found here are unexpected. Thus, their existence poses a challenge to theories.

The soft-core potentials employed here have considerable theoretical merit^33^, which can also mimic particle interactions in low-density experimental systems such as poly *N*-isopropylacrylamide colloids, granular materials, and foams^34–36^. How to extend the repulsions to long range and hence reach the high-density regime is challenging to the experimental verification of our findings. Possible solutions are to use ultrasoft particles such as star polymers^37^ or to modulate long-range repulsive interactions of colloidal or granular systems composed of magnetic or charged particles^38^.

One of the most special features of our QCs is the spontaneous formation of pentagons, to some extent similar to ABC star polymer QCs^5^ and cluster QCs^39^. This leads to a complex structural unit: a pentagon surrounded by an *n*-side polygon. The spontaneous formation of this structural unit should not be an accident, because it occurs in all our QCs with different rotational symmetries. Therefore, our findings suggest a promising motif to design *n*-fold QCs. A recent experimental work has shown that in two-dimensional solid states of magnetic polygons, the vertexes of the polygons can display weak 12-fold rotational symmetry (manuscript in preparation). This may imply the possibility to form two-dimensional QCs in granular systems consisting of magnetic pentagons and small disks, which deserves further investigations.

## Methods

### Systems and simulation details

Our systems are two- or three-dimensional boxes with side length *L*. Periodic boundary conditions are applied in all directions. The system contains *N* monodisperse particles with a mass *m*. The units of energy, length, and mass are $\epsilon$, *σ*, and *m*. The time and temperature are in units of *m* ^1/2^ *σ* $\epsilon$ ^−1/2^ and $\epsilon k_{\mathrm{B}}^{ - 1}$ with *k*_B_ being the Boltzmann constant. In this work, we mainly study *N* = 10,000 and 4096 systems.

We perform molecular dynamics (MD) simulations in both the *NVT* (constant number of particles, volume, and temperature) and *NPT* (constant number of particles, pressure, and temperature) ensembles. To outline the phase diagram, we slowly quench high-temperature liquids until solids are formed. We have verified that the quench rates are slow enough so that the phase boundaries are not sensitive to the change of the quench rate. To make sure that systems are in equilibrium, we first relax the system for a long time (5 × 10^9^ MD steps with a time step Δ*t* = 0.01 for solid states and 10^8^ MD steps for liquid states) and then collect data in the following 10^8^ MD steps. To get static configurations at *T* = 0, we directly quench the equilibrium solid states to local potential energy minima using the fast inertial relaxation engine algorithm^40^.

### Structural and dynamical quantities

For two-dimensional systems, the diffraction patterns and density profiles are calculated from the static structure factor and radial distribution function, respectively: $S({\bf{k}}) = \frac{1}{N}\left\langle {\rho ({\bf{k}})\rho ( - {\bf{k}})} \right\rangle$ and $g({\bf{r}}) = \frac{{L^2}}{{2N^2}}\left\langle {\mathop {\sum}\nolimits_{i = 1}^N \mathop {\sum}\nolimits_{j \ne i}^N \delta \left( {{\bf{r}} - {\bf{r}}_{ij}} \right)} \right\rangle$, where $\rho ({\bf{k}}) = \mathop {\sum}\nolimits_{j = 1}^N e^{{i}{\bf{k}} \cdot {\bf{r}}_j}$ is the Fourier transform of the density with **r**_*j*_ being the location of particle *j*, **k** is the wave vector satisfying the periodic boundary conditions, **r**_*ij*_ = **r**_*i*_ −**r**_*j*_ is the separation between particles *i* and *j*, the sums are over all particles, and $\left\langle . \right\rangle$ denotes the time average. The van Hove autocorrelation function is calculated from $G_{\mathrm{a}}({\bf{r}},t) = \left\langle {\frac{1}{N}\mathop {\sum}\nolimits_i \delta \left[ {{\bf{r}} - {\bf{r}}_i(t) + {\bf{r}}_i(0)} \right]} \right\rangle$, where $\left\langle . \right\rangle$ denotes the ensemble average and the sum is over all particles. *S*(*k*) and *g*(*r*) are quantities averaged over all directions of **k** and **r** with $k = \left| {\bf{k}} \right|$ and $r = \left| {\bf{r}} \right|$.

### Calculation of the perpendicular space

In the physical space where QCs reside, every particle is actually the center of a cluster formed by this particle and its nearest neighbors. To obtain particle (cluster center) projections in the perpendicular space, we lift particles from the two-dimensional physical space to the four-dimensional hyperspace using appropriate basis vectors and then perform the projection. The basis vectors in the physical (parallel) space are ${\bf{e}}_{{\mathrm{OQC}}}^{{\mathrm{||}}} = \frac{1}{{\sqrt 2 }}({\mathrm{cos}}(2\pi i{\mathrm{/}}8),{\mathrm{sin}}(2\pi i{\mathrm{/}}8))$ and ${\bf{e}}_{{\mathrm{DDQC}}}^{{\mathrm{||}}} = \frac{1}{{\sqrt 3 }}({\mathrm{cos}}(2\pi i{\mathrm{/}}12),{\mathrm{sin}}(2\pi i{\mathrm{/}}12))$ (*i* = 0, …, 3) for OQCs and DDQCs, respectively. In the perpendicular space, there are corresponding basis vectors ${\bf{e}}_{{\mathrm{OQC}}}^ \bot$ and ${\bf{e}}_{{\mathrm{DDQC}}}^ \bot$ orthogonal to ${\bf{e}}_{{\mathrm{OQC}}}^{||}$ and ${\bf{e}}_{{\mathrm{DDQC}}}^{{\mathrm{||}}}$. Having selected an arbitrary particle in the QC as the origin, the location of this particle in the physical space is transformed to ${\bf{r}}^{{\mathrm{||}}} = \mathop {\sum}\nolimits_{i = 0}^3 n_i{\bf{e}}_i^{||}$, where *n*_*i*_ is an integer index determined by nearest neighbors: *n*_*i*_ is increased or decreased by 1 if a vector connecting the particle and a neighbor is parallel or antiparallel to ${\bf{e}}_i^{||}$, and 0 otherwise^17^. The particle’s coordinates in the four-dimensional hyperspace is thus (*n*_0_, *n*_1_, *n*_2_, *n*_3_). Its projection in the perpendicular or parallel space is ${\bf{r}}^ \bot = \mathop {\sum}\nolimits_{i = 0}^3 n_i{\bf{e}}_i^ \bot$ or **r** ^||^. If a particle exhibits the quasicrystalline order, its projection in the perpendicular space will fall within the atomic surface, which is the projection of the unit cell of the hyperspace in the perpendicular space^23^.

### Data availability

The data that support the findings of this study are available from the corresponding author upon reasonable request.

## Electronic supplementary material

Supplementary Information

Description of Additional Supplementary Files

Supplementary Movie 1

Supplementary Movie 2
