# Supplementary material for: Forming quasicrystals by monodisperse soft core particles
Source: Nat Commun. 2017 Dec 12;8:2089. doi: 10.1038/s41467-017-02316-3 (PMC5727032; doi:10.1038/s41467-017-02316-3)
Supplement: Supplementary file 1 — Supplementary Information [file 41467_2017_2316_MOESM1_ESM.pdf]

Supplementary Figures

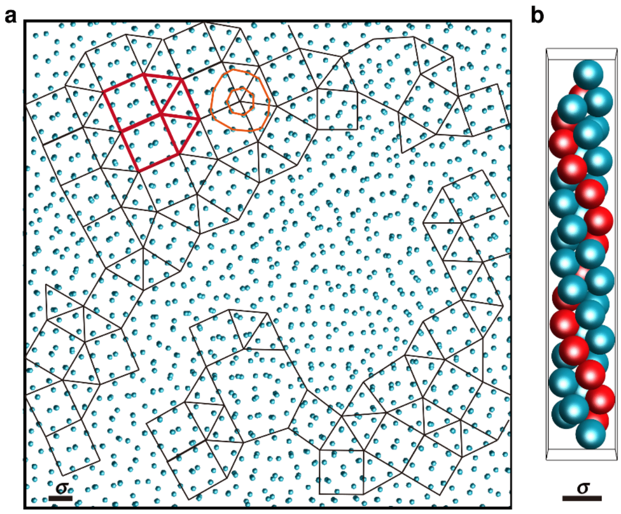

Supplementary Figure 1. Characterization of structure of a solid with Archimedean tiling in three dimensions at $(\rho, \alpha, T = 3.6, 2.5, 1.0 \times 10^{-4})$. **a** A part of static configuration viewed from the top of parallel tubes with 7-fold rotational symmetry. The centers of the heptagons are connected to form the square-triangle tessellation partially. The red lines outline the typical $(3^3.4^2)$ Archimedean tiling. The orange loops outline the structure with a heptagon (inner loop) surrounded by a dodecagon (outer loop). **b** Side view of a single tube with 7-fold symmetry. Particles exhibit clear chirality, as illustrated by the red particles. In both **a** and **b**, the black scale bar indicates the actual size of particle diameter σ.

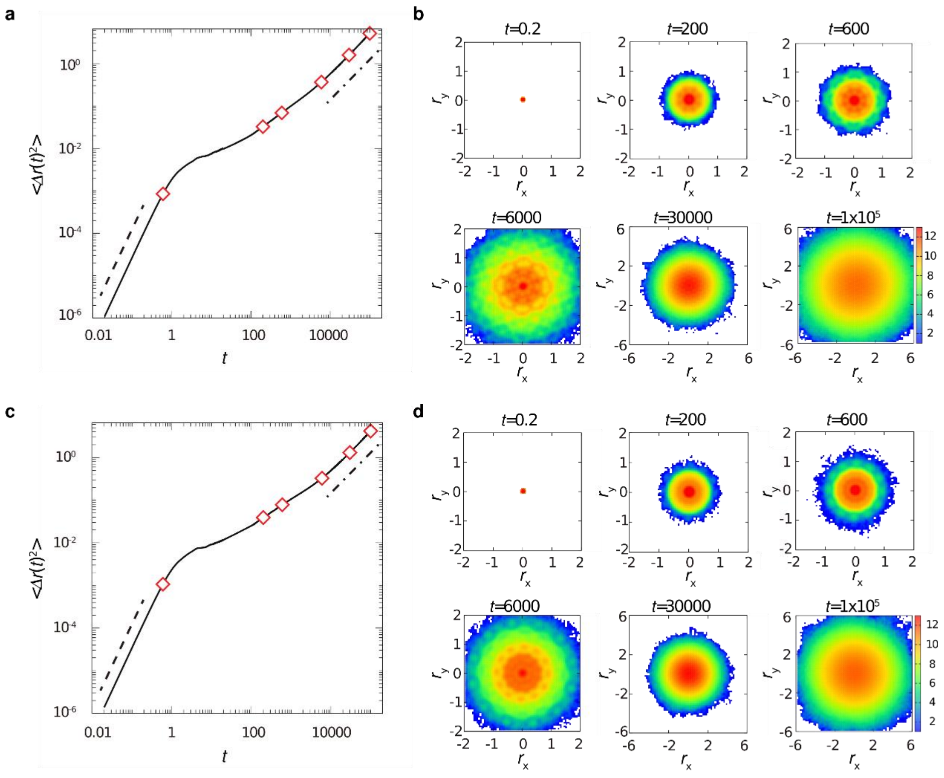

Supplementary Figure 2. Dynamics of QCs. **a**, **b** Mean square displacement, $\langle \Delta r(t)^2 \rangle$, and time evolution of the van Hove autocorrelation function $G_\mathrm{a}(\mathbf{r}, t)$ for a OQC at $(\alpha, \rho) = (2.0, 6.60)$. **c** and **d** are for a DDQC at $(\alpha, \rho) = (2.0, 7.00)$. The dashed and dot-dashed lines in **a** and **c** have a slope of 2 and 1, respectively. The squares in **a** and **c** label the instants for which $G_\mathrm{a}(\mathbf{r}, t)$ are shown in **b** and **d**. The scale bars show values of G_a in the logarithmic scale.

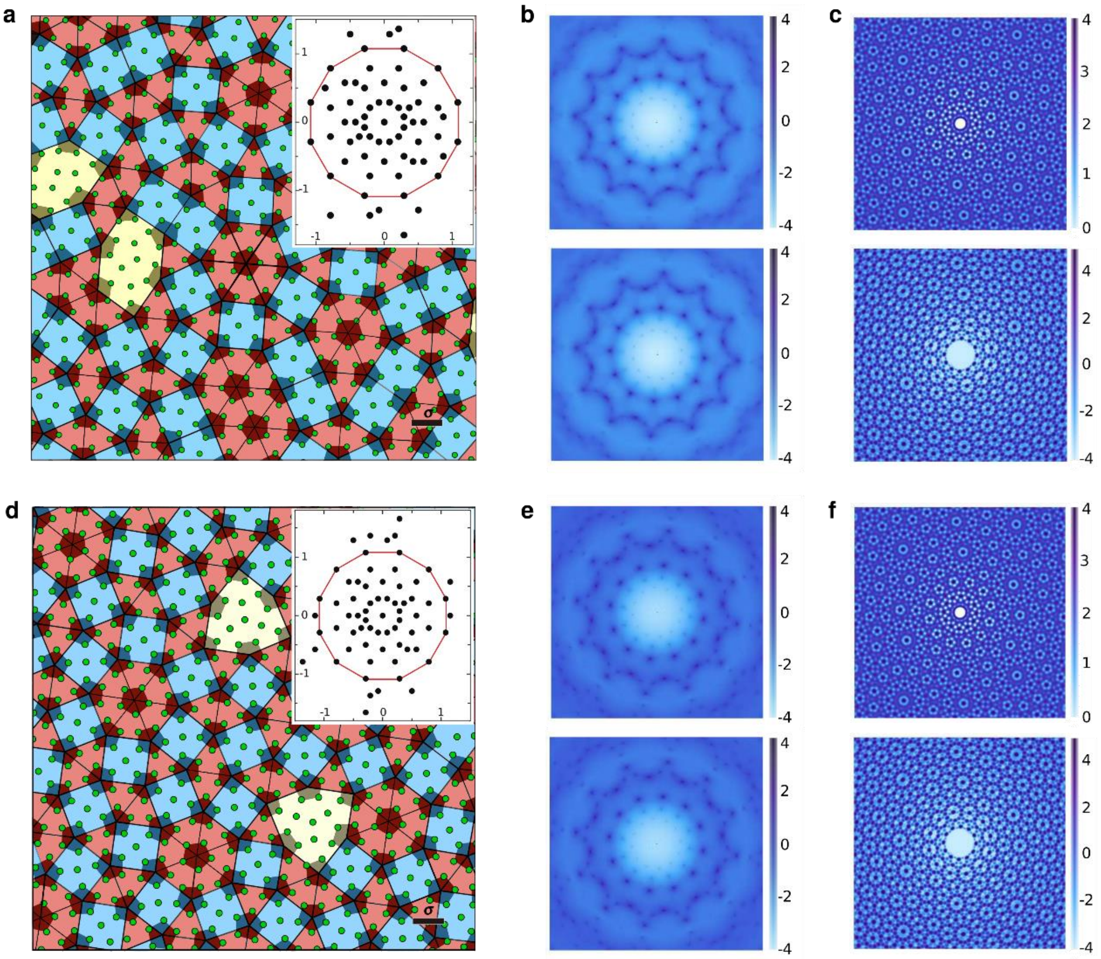

Supplementary Figure 3. Characterization of structure of LDDQCs. **a**-**c** and **d**-**f** are for DDQCs at $(\rho, \alpha, T) = (2.56, 2.0, 1.53 \times 10^{-3})$ and $(3.46, 2.5, 6.80 \times 10^{-4})$, respectively. **a** (**d**) A part of static configuration with the square-triangle tiling. The black scale bar indicates the actual size of particle diameter σ. Note that shadowed pentagons prevail, whose centers are connected to construct the tessellation. The inset shows the projection of the QC in the perpendicular space with the red dodecagon being the atomic surface. **b, c** (**e, f**) Diffraction patterns and density profiles calculated from single particles [top panel] and from pentagons [bottom panel]. The values of the scale bars are in the logarithmic scale.

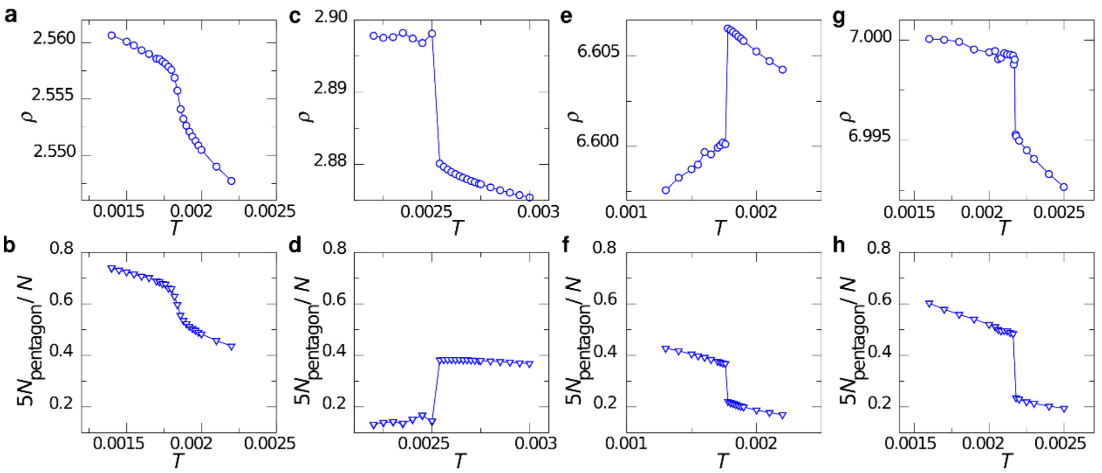

Supplementary Figure 4. Temperature dependence of density and number of pentagons across the liquid-solid phase transitions for systems with $\alpha = 2.0$. The top and bottom rows show density, $\rho(T)$, and fraction of particles forming pentagons, $5N_{\text{pentagon}}(T)/N$, respectively. From the left to the right, the solid phases are LDDQC (**a** and **b**), stretched honeycomb (**c** and **d**), OQC (**e** and **f**), and HDDQC (**g** and **h**).

Supplementary Notes

Supplementary Note 1: Preliminary results of three-dimensional systems.

In the main text, we concentrate on two-dimensional (2D) systems. It is interesting to know whether QCs or related phases exist in three-dimensional (3D) systems of the same model. In this section, we show some preliminary results of 3D systems. Compared with 2D systems, the inclusion of a lot more degrees of freedom increases the complexity of the phase formation in 3D. As will be shown below, we do find interesting phases in 3D, which call for further in-depth studies.

Inspired by 2D results, we guess that if 3D QCs exist they may also reside adjacent to (on higher density side of) solids with square lattice structure in certain planes, such as BCC solids. We then focus on density regimes next to BCC solids for both $\alpha = 2.0$ and 2.5. Here we use exactly the same slow-quenching protocol as introduced in the section of Methods of the main text. In some narrow parameter regimes, we find the formation of parallel tubes with 7-fold rotational symmetry. In the top view of the tubes (Supplementary Fig. 1a), we can identify many heptagons. Each heptagon is surrounded by a dodecagon, forming a complicated structural unit similar to those in 2D QCs. By connecting centers of non-edge-adjacent heptagons, we obtain a partial square-triangle tiling as for the 2D DDQCs. Interestingly, it is the $(3^3.4^2)$ Archimedean tiling. It has been shown that the Archimedean tiling is closely related to QCs. For example, it could occur between quasicrystalline and crystalline phases[1] and could develop into QCs with the modulation of parameters[2,3]. Probably because we have not found appropriate parameters, we have not observe convincing QCs in 3D. However, the observation of Archimedean tiling in our 3D systems is extraordinary. It indicates the great possibility to obtain 3D QCs in our simple systems. Moreover, to our knowledge, the observation of Archimedean tiling in 3D systems has been rarely reported. The formation of Archimedean tiling observed here thus deserves further studies.

Supplementary Fig. 1b shows another interesting feature of the 3D system. The side view of a single tube with 7-fold symmetry reveals the emergence of chirality. The chiral packing of particles may cause high energy barriers for QCs to form. The formation of the chirality and its role in the formation of complicated structures observed here or even QCs are interesting issues to attack in follow-up studies.

Supplementary Note 2: Dynamics of QCs.

As discussed in the main text, particles in QCs show long-time diffusive motion caused by a sequence of phason flips[4-8]. In Supplementary Fig. 2, we show the particle mean squared displacement $\langle \Delta\, r(t)^2 \rangle$ and van Hove autocorrelation function $G_{\mathrm{a}}(\mathbf{r}, t)$ calculated at different times for both OQCs and DDQCs. We also provide movies (Supplementary Movie 1 and Supplementary Movie 2) with a total recording time of 10^5, from which the real-time trajectories of the tagged particles are visualized. From Supplementary Fig. 2, we can clearly see the short-time ballistic ($\langle \Delta\, r(t)^2 \rangle \sim t^2$) and long-time diffusive ($\langle \Delta\, r(t)^2 \rangle \sim t$) behaviors. In between, there is a sub-diffusive regime. The QC symmetry can be clearly identified from $G_{\mathrm{a}}(\mathbf{r}, t)$ in the intermediate time regimes before the long-time diffusion.

Supplementary Note 3: Structure of LDDQCs.

In the main text, we mainly show results of QCs at higher densities. In Supplementary Fig. 3, we characterize the structure of LDDQCs (lower-density DDQCs) for both $\alpha = 2.0$ and 2.5. The LDDQCs exhibit the same structural features as the higher-density ones, e.g., 12-fold rotational symmetry, formation of pentagons, and square-triangle tessellation, only that there are more defects at lower densities.

Supplementary Note 4: Phase transition from liquids to QCs.

In Supplementary Fig. 4, we show the temperature dependence of the density, $\rho(T)$, and the fraction of particles forming pentagons, $5N_{\mathrm{pentagon}}(T)/N$, across the melting temperature T_{m} in the *NPT* ensemble for the $\alpha = 2.0$ QCs at different pressures. We also show a case for the lower-density stretched honeycomb (LSHon) for comparison. For the system sizes studies in this work, OQCs and DDQCs at higher densities exhibit discontinuous phase transitions, while the phase transition of DDQCs at lower densities still looks more or less continuous with a fast change in the vicinity of the transition. Although the nature of the phase transition may depend on density for soft-core systems in two dimensions[9], much larger systems and finite size analysis are required to determine the nature of the liquid-QC transition in follow-up studies.

As stressed in the main text, pentagons are essential in the formation of our QCs. As shown in Supplementary Fig. 4, $5N_{\mathrm{pentagon}}(T)/N$ jumps to a much larger value than that in liquid when phase transition to QCs happens. In contrast, although there are lots of pentagons in the liquid states, when LSHons (adjacent to LDDQCs as shown in Fig. 1a of the main text) are formed, $5N_{\mathrm{pentagon}}(T)/N$ drops to a small value. Note that in Fig. 4 of the main text, $5N_{\mathrm{pentagon}}(T)/N$ exhibits broad peaks in the density regimes where two competing length scales emerge in the static structure factor $S(k)$. $5N_{\mathrm{pentagon}}(T)/N$ reaches the maximum for QC-forming liquids, which we believe is not a coincidence. Now we see from Supplementary Fig. 4 that the fate of pentagons in

QC-forming liquids is indeed different from that in other liquids: The pentagons survive and grow across the phase transition to QCs, while mostly annihilated in non-quasicrystalline liquid-solid transitions. It is thus interesting to investigate in follow-up studies whether the origins and roles of pentagons in the broad peaks of $5N_{\mathrm{pentagon}}(T)/N$ in Fig. 4 of the main text are different for QC-forming liquids from others.

Supplementary References

1. Talapin, D. V. *et al.* Quasicrystalline order in self-assembled binary nanoparticle superlattices. *Nature* **461**, 964-967 (2009).
2. Mikhael, J., Roth, J., Helden, L. & Bechinger, C. Archimedean-like tiling on decagonal quasicrystalline surfaces. *Nature* **454**, 501-504 (2008).
3. Schmiedeberg, M. *et al.* Archimedean-like colloidal tilings on substrates with decagonal and tetradecagonal symmetry. *Eur. Phys. J. E* **32**, 25-34 (2010).
4. Janot, C. Quasicrystals: A Primer. (Oxford Uni. Press, 1997).
5. Lubensky, T. C. & Ramaswamy, S. Hydrodynamics of icosahedral quasicrystals. *Phys. Rev. B* **32**, 7444-7452 (1985).
6. Ashraff, J. A., Luck, J-M. & Stinchcombe, R. B. Dynamical properties of two-dimensional quasicrystals. *Phys. Rev. B* **41**, 4314-4329 (1990).
7. Hocker, S. & Gähler, F. Aluminium diffusion in decagonal quasicrystals. *Phys. Rev. Lett.* **93**, 075901 (2004).
8. Engel, M., Umezaki, M., & Trebin, H-R. Dynamics of particle flips in two-dimensional quasicrystals. *Phys. Rev. E* **82**, 134206 (2010).
9. Zu, M. J., Liu, J., Tong, H. & Xu, N. Density Affects the Nature of the Hexatic-Liquid Transition in Two-Dimensional Melting of Soft-Core Systems. *Phys. Rev. Lett.* **117**, 085702 (2016).
