# Supplementary material for: Forming quasicrystals by monodisperse soft core particles
Source: Nat Commun. 2017 Dec 12;8:2089. doi: 10.1038/s41467-017-02316-3 (PMC5727032; doi:10.1038/s41467-017-02316-3)
Supplement: Supplementary file 2 — Description of Additional Supplementary Files [file 41467_2017_2316_MOESM2_ESM.pdf]

Description of Supplementary Files

File Name: Supplementary Movie 1
Description: Time evolution of an octagonal quasicrystal over a total recording time of 10^5. The orange particles are tagged particle to illustrate the single particle motion.

File Name: Supplementary Movie 2
Description: Time evolution of a dodecagonal quasicrystal over a total recording time of 10^5. The orange particles are tagged particles to illustrate the single particle motion.
